# Development of Gene-Based SSR Markers in Rice Bean (*Vigna umbellata* L.) Based on Transcriptome Data

**DOI:** 10.1371/journal.pone.0151040

**Published:** 2016-03-07

**Authors:** Honglin Chen, Xin Chen, Jing Tian, Yong Yang, Zhenxing Liu, Xiyu Hao, Lixia Wang, Suhua Wang, Jie Liang, Liya Zhang, Fengxiang Yin, Xuzhen Cheng

**Affiliations:** 1 The National Key Facility for Crop Gene Resources and Genetic Improvement, Institute of Crop Science, Chinese Academy of Agricultural Sciences, Beijing 100081, China; 2 Institute of Vegetable Crops, Jiangsu Academy of Agricultural Sciences, Nanjing 210014, Jiangsu, China; 3 Institute of Cereal and Oil Crops, Hebei Academy of Agricultural and Forestry Sciences, Shijiazhuang 050035, Hebei, China; 4 Crop Research Institute, Anhui Academy of Agricultural Sciences, Hefei 230031, Anhui, China; 5 Tangshan Academy of Agricultural Sciences, Tangshan 036001, Hebei, China; 6 Baicheng Academy of Agricultural Sciences, Baicheng 137000, Jilin, China; Università Politecnica delle Marche, ITALY

## Abstract

Rice bean (*Vigna umbellata* (Thunb.) Ohwi & Ohashi) is a warm season annual legume mainly grown in East Asia. Only scarce genomic resources are currently available for this legume crop species and no simple sequence repeat (SSR) markers have been specifically developed for rice bean yet. In this study, approximately 26 million high quality cDNA sequence reads were obtained from rice bean using Illumina paired-end sequencing technology and assembled into 71,929 unigenes with an average length of 986 bp. Of these unigenes, 38,840 (33.2%) showed significant similarity to proteins in the NCBI non-redundant protein and nucleotide sequence databases. Furthermore, 30,170 (76.3%) could be classified into gene ontology categories, 25,451 (64.4%) into Swiss-Prot categories and 21,982 (55.6%) into KOG database categories (E-value < 1.0E-5). A total of 9,301 (23.5%) were mapped onto 118 pathways using the Kyoto Encyclopedia of Genes and Genome (KEGG) pathway database. A total of 3,011 genic SSRs were identified as potential molecular markers. AG/CT (30.3%), AAG/CTT (8.1%) and AGAA/TTCT (20.0%) are the three main repeat motifs. A total of 300 SSR loci were randomly selected for validation by using PCR amplification. Of these loci, 23 primer pairs were polymorphic among 32 rice bean accessions. A UPGMA dendrogram revealed three major clusters among 32 rice bean accessions. The large number of SSR-containing sequences and genic SSRs in this study will be valuable for the construction of high-resolution genetic linkage maps, association or comparative mapping and genetic analyses of various *Vigna* species.

## Introduction

There are currently 21 species in the genus *Vigna* subgenus *Ceratotropis* that are distributed across a wide region of Asia. Six cultivated species belong to this subgenus. Mung bean (*V*. *radiata*), adzuki bean (*V*. *angularis*), rice bean (*V*. *umbellata)*, and black gram (*V*. *mungo*) are the four most economically important cultivated species in Asian countries. Rice bean (*Vigna umbellata* (Thunb.) Ohwi & Ohashi) is a short-day, warm-season annual legume that is cultivated mainly in Nepal, Bhutan, northeast India, Myanmar, southern China, northern Thailand, Laos, Vietnam, Indonesia and East Timor [1].

Rice bean has a high yield potential and good nutritional quality. It is mainly grown for human consumption and is also important as a fodder, green manure and vegetable. It has the potential to produce large amounts of nutritious animal fodder and high quality grain. In recent years, rice bean has been sometimes substituted for adzuki bean (*Vigna angularis* (Willd.) Ohwi & Ohashi) in the making of pastry [1].

Molecular markers have proven to be an important and effective tool for germplasm evaluation, genetic diversity analysis, gene mapping and marker-assisted selection (MAS) for crop improvement. However, genomic studies of this crop has lagged behind that of other legume crops, such as soybean, common bean, mung bean, and adzuki bean due to the lack of polymorphic DNA molecular markers. To date, there are not many molecular markers reported for rice bean, and only very few of them, such as RFLP [2], RAPD [3], AFLP [4], and ISSR [3] have been reported in rice bean, particularly microsatellites or simple sequence repeat markers. SSRs are an ideal choice for broadening the scope of the markers available to these species researchers because of their abundance, high polymorphism, codominance, reproducibility, and transferability to related legumes species.

In contrast to genomic SSRs, genic SSRs are located in the coding region of the genome and have some intrinsic advantages. For an example, they are in more conserved coding regions of the genome, and the generation of genic SSR markers is relatively easy and inexpensive, and highly transferable to related taxa. Because of these advantages, genic SSRs have been developed and used in many plant species. There are no reports on SSR markers for rice bean to date. They have only been developed from related *Vigna* species, such as mung bean [5] and adzuki bean [1]. Thirteen SSR markers developed for adzuki bean, which have been distributed across its 11 chromosomes were used to assess the genetic diversity of rice bean [5]. Forty-seven SSR markers developed for mung bean showed reliable banding and polymorphisms were used to analyze the genetic diversity of 230 rice bean accessions [1]. The number of SSR markers used in rice bean so far is fewer than those reported for other legumes, such as mung bean [6], adzuki bean [7], common bean [8, 9], chickpea [10], pigeon pea [11] and soybean [12, 13].

In this study, we generated a large number of high-quality rice bean transcriptome sequences and developed a large number of gene-based SSRs using the Illumina HiSeq sequencing platform. The distribution of SSR motifs in the sequences generated was characterized, and a set of genic SSR for further use in diversity analysis was validated.

## Materials and Methods

### Plant materials

In total, thirty-two accessions of rice bean collected from various parts of China were used to analyze the genetic diversity in this study (S1 Table). These rice bean accessions were obtained from the National Center for Crop Germplasm Resources Preservation at the Institute of Crop Science, Chinese Academy of Agricultural Sciences (ICS-CAAS), Beijing, China. ICS-CAAS is responsible for the collection of rice bean accessions. One variety, ‘Jingfan No. 1’ which originates from Beijing, China, was used for RNA-seq. Roots, leaves, and shoots were collected from five individual rice bean plants.

### RNA isolation and cDNA library construction

Total RNA was isolated from the roots, stems, and leaves of five plants of the rice bean variety ‘Jingfan No. 1’ using Trizol Reagent, according to the manufacturer’s instructions (Invitrogen, Carlsbad, CA, USA). The RNA was then treated with RNase-free DNase I (Takara, Otsu, Shiga, Japan) at 37°C for 30 min to remove residual DNA. The RNA quality was verified using a 2100 Bioanalyzer (Agilent Technologies, Santa Clara, CA) and confirmed using RNase-free agarose gel electrophoresis. The concentration of the total RNA was further quantified using NanoDrop 2000 (Thermo Fisher Scientific, Wilmington, Delaware USA). Equal amounts of total RNA from rice bean ‘Jingfan No. 1’ were quickly frozen in liquid nitrogen for storage at -80°C until further use.

A cDNA library of pooled RNA was obtained using a TruSeq RNA Sample preparation kit (Illumina, USA), according to the manufacturer's instructions. Poly-T oligo-attached magnetic beads (Illumina Inc., San Diego USA) were used to isolate poly-A mRNA from total RNA. First-strand cDNA was synthesized from the fragmented mRNA using random hexamer primers and reverse transcriptase (Invitrogen, USA). The single-end cDNA library was prepared according to Illumina’s recommended protocol.

### Illumina Sequencing, data filtering and *de novo* assembly

The cDNA library was sequenced using PE90 strategy with Illumina paired-end sequencing technology according to the standard Illumina protocol of Biomarker Technologies Co., Ltd (Beijing, China). The libraries were sequenced in one lane, and raw-reads were subsequently sorted using barcodes. The data was automatically collected and generated into FASTQ files (.fq) containing raw data for all reads. The data was submitted to the Sequence Read Archive (SRA) of the NCBI database under accession number SRX1050017. The raw sequence data were first cleaned using trimming adapter sequences. Reads containing more than 10% of bases with a poor quality score (Q<20), non-coding RNA, ambiguous sequences containing an excess of “N” nucleotide calls or adaptor contamination were removed. Reads that did not pass the Illumina failed-chastity filter were discarded, according to “failed-chastity ≤ 1” with a threshold of 0.6 on the first 25 cycles. *De novo* transcriptome assembly was then performed using Trinity [14].

### Unigene annotation

The unigenes were aligned with BLASTX against four protein databases (NCBI non-redundant or Nr proteins, Swiss-Prot, Kyoto Encyclopedia of Genes and Genomes or KEGG and euKaryotic Ortholog Groups or KOG) and one nucleotide database (NCBI nucleotide or Nt sequences) with an E-value threshold of 1.0E-5 for all except KOG with a threshold of 1.0E-3 [15, 16]. Using nucleotide-based annotation, Blast2GO [17] software was used to obtain GO annotation categories defined by molecular function, cellular component and biological process ontologies.

### SSR search and primer design

MISA (MIcroSAtellite; http://pgrc.ipk-gatersleben.de/misa) and SAMtools [18] were employed for SSR mining and identification. The minimum number of repeats used to select the SSRs was ten for mono-nucleotide repeats, six for di-nucleotide repeats, five for tri-nucleotide repeats, and three for tetra-, penta-, and hexa-nucleotide repeats. Primers for SSRs were designed using Premier 5.0 (PREMIER Biosoft International, Palo Alto, California, USA) with the following criteria: primer lengths of 16–22 bases, GC content of 40–60%, annealing temperature of 40°C–60°C, and PCR product size of 100 to 300 bp.

### Marker validation

A total of 300 genic SSR markers were validated using 32 rice bean accessions. Genomic DNA of each rice bean accession was extracted from young leaves using the Hexadecyl trimethyl ammonium bromide (CTAB) method [19]. The quality and quantity of DNA was evaluated on a 1% agarose gel. The DNA concentration was adjusted to 50 ng/μl for SSR marker analysis. PCR was performed in a total volume of 20 μl containing 50 ng of genomic DNA, 0.5 U of Taq DNA polymerase (Dingguo Biological Technology Development Co., Ltd, Beijing, China), 1× of Taq Buffer II, 1.5 mM MgCl_2_, 25 μM of dNTPs, and 0.4 μM each forward and reverse primer. PCR amplification was performed using a Heijingang Thermal Cycler (Eastwin, Beijing, China) with the following cycling conditions: pre-denaturation at 94°C for 4 min followed by 30–35 cycles of 94°C for 30 sec, 55–60°C (depending on primers) for 30 sec and 72°C for 30 sec, and finally, 5 min at 72°C. The PCR products were separated on an 8.0% non-denaturing polyacrylamide gel electrophoresis (PAGE) gel and then visualized by silver staining. The pBR322 Marker I DNA ladder (Zheping, Biological Technology Development Co., Ltd, Beijing, China) was used as the standard size marker.

### Genetic diversity analysis

The number of alleles (Na), expected heterozygosity (He), gene diversity, and polymorphism information content (PIC) for each of the genic SSR markers were calculated using PowerMarker V3.25 [20]. A genetic similarity matrix based on the “proportion of shared alleles” among the 32 rice bean accessions was generated using PowerMarker. An Unweighted Pair Group Method with Arithmetic Mean (UPGMA) tree based on the shared allele distances was constructed using MEGA 4 software [21] to reveal genetic relationships between the 32 rice bean accessions.

## Results

### Sequencing and *de novo* assembly of Illumina paired-end reads

**Fig 1:**
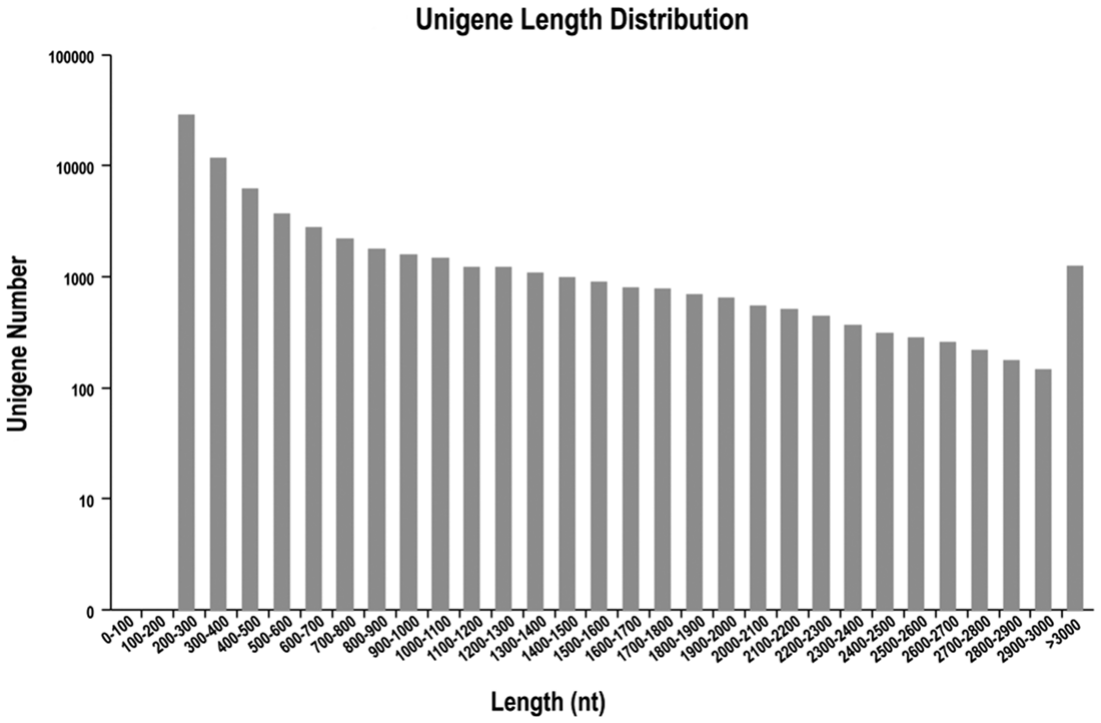
Frequencies length distribution of Illumina read sequences.

Approximately 25.95 million clean reads with a GC content of 47.87% was obtained for the ‘Jingfan No. 1’ variety. The sequence quality, based upon the clean reads of both rice bean varieties, was 94.1% of Q20. The combined sequences of these reads were assembled into 71,929 unigenes and 122,645 individual transcripts. The average length of the assembled unigenes was 986 bp, with N50 = 1,677 bp, which was longer than the average length of the assembled transcripts (668 bp, N50 = 1,144 bp). Of these unigenes, 45,898 (63.8%) were 201 to 500 bp; 11,945 (16.6%) were 501 to 1,000 bp; 5,876 (8.2%) were 1,001 to 1,500 bp; 3,789 (5.3%) were 1,501 to 2,000 bp; 2,142 (3.0%) were 2,001 to 2,500 bp; 1,061 (1.5%) were 2,501 to 3,000 bp; and 1,218 (1.7%) were more than 3,000 bp in length (Fig 1).

### Sequence annotation

For annotation of the sequence assembly contigs and unique singletons, all unigenes were searched against the five databases (see Materials and methods). A total of 39,530 unigenes showed significant BLAST hits. Among these unigenes, 38,840 (98.3%) showed significant similarity to known proteins in the NR sequence database, of which 25,451 (64.4%) were similar to protein in the Swiss-Prot.

**Fig 2:**
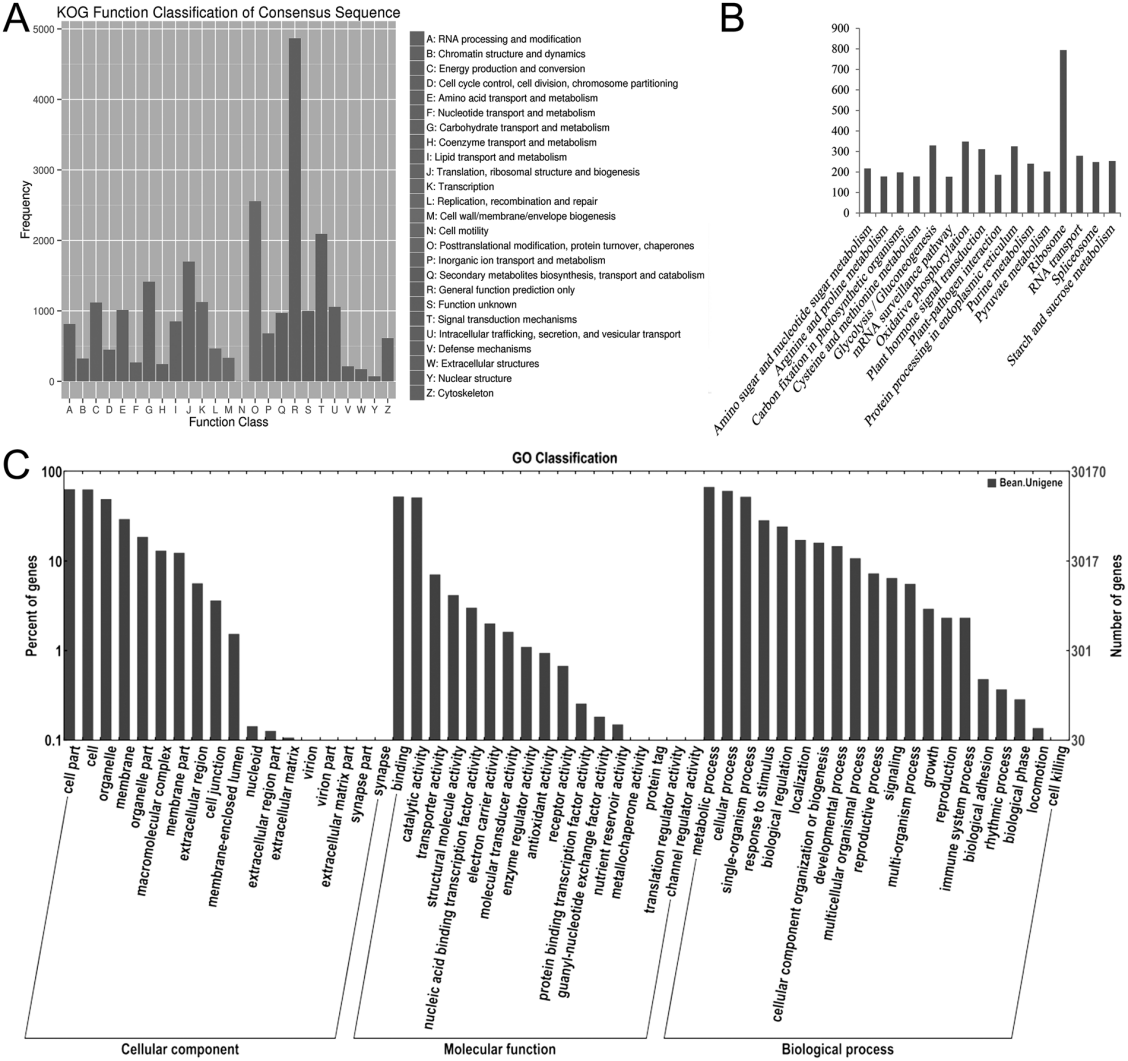
Classification of assembled unigenes. (A) EuKaryotic Ortholog Groups (KOG) classification of assembled unigenes. (B) Kyoto Encyclopedia of Genes and Genomes (KEGG) classification of assembled unigenes. (C) Gene ontology (GO) classification of assembled unigenes

Using KOG functional classification, 21,982 (55.6%) unigenes aligned to the KOG database and were classified into 25 functional categories, among which the general function prediction was the largest group (3,140 unigenes, 17.4%), followed by replication, recombination and repair (1,703, 9.4%), translation, ribosomal structure and biogenesis (1,612, 8.9%), and transcription (1,482, 8.2%). Cell motility (22, 0.1%) and nuclear structure (11, 0.1%) represented the smallest groups predicted by KOG (Fig 2A).

To characterize the active biological pathways in rice bean, we used the Kyoto Encyclopedia of Genes and Genomes (KEGG) to analyze the pathway annotations of unigene sequences. In total, 9,301 (23.5%) unigenes were annotated to 118 pathways by searching the KEGG pathway database. The most highly represented pathways were ribosome (794 unigenes, 7.5%), oxidative phosphorylation (348, 3.3%), and glycolysis (329, 3.1%). Caffeine metabolism (2, 1.9%) and glucosinolate biosynthesis (1, 0.9%) represented the smallest groups (Fig 2B).

On the basis of the annotation against the NR database, 30,170 (76.3%) unigenes were assigned to gene ontology (GO) terms. Sequences that belonged to the biological process, cellular component and molecular function clusters were categorized into 55 terms. Within the cellular component category, cell component (19,120, 24.3%) and organelle component (14,837, 18.9%) represented the majorities, whereas only a few unigenes were assigned to extracellular matrix (6, <0.1%) and synapse (1, <0.1%). Within the biological process category, the highest sub-category was metabolic process (20,281, 21.0%), followed by cellular process (18,363, 19.0%), and single-organism process (15,757, 16.3%). Under the molecular function category, binding activity (15,837, 42.0%) and catalytic activity (15,475, 41.0%) were prominently represented. Furthermore, 2,637 unigenes were involved in transporter activity, whereas only a few unigenes were assigned to protein tag (7), translation regulator activity (6) and channel regulator activity (1) (Fig 2C).

### Frequency and distribution of different SSR types

Of the 71,929 unigenes generated in the study, 3,011 SSRs were identified (S2 Table). A total of 3,868 unigenes contained SSR, 634 sequences had more than one SSR, and 185 had SSRs of different motifs (compound SSRs). The proportion of SSRs was not evenly distributed; the mono-nucleotide repeat motifs were the most abundant (1,831 or 47.3%), followed by di- (1,038 or 26.8%), tri- (955 or 24.7%), tetra- (30 or 0.8%), penta- (9 or 0.2%) and hexa-nucleotide (5 or 0.1%) repeat motifs (Table 1).

**Table 1:** Summary of the number of repeat units in rice bean SSR loci.

Repeat motif	No. of repeats							
	5	6	7	8	9	10	>10	Total
Mono-nucleotide (1,831)								
A/T	-	-	-	-	-	905	915	1,820
C/G	-	-	-	-	-	6	5	11
Di-nucleotide (1,038)								
AG/CT	-	106	72	53	35	29	20	315
AT/TA	-	62	46	20	23	16	16	183
AC/GT	-	19	8	8	1	-	-	36
Others	-	253	145	104	2	-	-	504
Tri-nucleotide (955)								
AAC/GTT	12	1	1	-	-	-	-	14
AAG/CTT	43	22	12	-	-	-	-	77
AAT/ATT	18	10	12	-	-	-	-	40
ACC/GGT	28	9	-	1	-	-	-	38
Others	497	196	92	-	-	-	1	786
Tetra-nucleotide (30)								
ATAA/TTAT	2	3	-	-	-	-	-	5
AGAA/TTCT	3	3	-	-	-	-	-	6
TATC/GATA	2	-	-	-	-	-	-	2
CACT/AGTG	1	1	-	-	-	-	-	2
Others	8	7	-	-	-	-	-	15
Penta-nucleotide (9)								
ACCAT/ATGGT	1	-	-	-	-	-	-	1
TTATA/TATAA	1	-	-	-	-	-	-	1
TTTTC/GAAAA	1	-	-	-	-	-	-	1
GAGAA/TTCTC	-	1	-	-	-	-	-	1
AACGC/GCGTT	1	-	-	-	-	-	-	1
Others	3	1	-	-	-	-	-	4
Hexa-nucleotide (5)								
GCACCA/TGGTGC	-	1	-	-	-	-	-	1
TTCTCT/AGAGAA	1	-	-	-	-	-	-	1
CTCAGC/GCTGAG	1	-	-	-	-	-	-	1
Others	2	-	-	-	-	-	-	2
Total	625	695	388	186	61	956	957	3,868
%	16.2	18.0	10.0	4.8	1.6	24.7	24.7	100

The number of SSR repeats ranged from 5 to 23, with ten repeats being the most abundant, followed by six and five repeats as the next most abundant. Motifs with more than 15 repeats were rare (6.6%). Among the nucleotide repeats, A/T (99.4%) was the most abundant. The other three major motifs were AG/CT (30.3%), AAG/CTT (8.1%), and AGAA/TTCT (20.0%) (Table 1).

### Development of polymorphic genic SSR markers

Three hundred SSR markers were randomly selected to test amplification and informative nature by analyzing a germplasm panel of 32 rice bean accessions (S3 Table). Of the 300 markers, 43 (14.3%) produced clear amplicons of the expected size, 21 (7.0%) amplified non-specific products, and 236 (78.7%) failed to amplify the DNA product. Of the successful markers, 23 (53.5%) showed polymorphisms in the 32 rice bean accessions. Among the polymorphic markers, 2, 14, 6 and 1 were mono-, di-, tri-, and tetra-nucleotide repeat markers, respectively. The polymorphism ratio of the genic SSR markers with di-, tri-, tetra-, penta-, and hexa-nucleotide repeats were 8.7%, 60.9%, 26.1% and 4.3%, respectively. The polymorphic markers detected 56 alleles in total with allele numbers varying between 2 and 5 and with a mean of 2.4 per marker.

### Gene functions of the unigene sequences containing polymorphic genic SSRs

To determine the possible functions of the 23 validated genic SSRs, they were subjected to BLASTn analysis with a non-redundant database of legume sequences. The results showed that most of the sequences were similar to known or hypothetical protein-encoding genes from soybean (*Glycine max* L.) and chromosome location information from adzuki bean (*Vigna angularis*) (S4 Table). Among the positive hits were genes for transcription factor TCP4-like and chaperone protein ClpB-like.

### Phylogenetic analysis of the cultivated rice bean accessions

The 23 polymorphic genic SSRs developed in this study were used to assess the genetic diversity and genetic relationships between the 32 rice bean accessions. These accessions were obtained across the geographic crop distribution in China. The expected heterozygosity (He) varied from 0 to 0.2258, with an average of 0.0945. Gene diversity ranged from 0.0605 (c25883.graph_c0) to 0.6411 (c24756.graph_c0) with an average of 0.3479. PIC values ranged from 0.0587 (c25883.graph_c0) to 0.5718 (c24756.graph_c0), with an average of 0.2898 (Table 2).

**Table 2:** Characteristics of the polymorphic SSR markers in 32 rice bean accessions.

SSR locus	Motif	Estimated allelic size (bp)	Na^a^	He^b^	PIC^c^
c9711.graph_c0	(A)16	224	3	0.1250	0.3589
c19803.graph_c1	(AAG)7	181	3	0.2258	0.3740
c21640.graph_c0	(ACG)7	103	2	0.1923	0.4484
c19719.graph_c0	(AG)11	209	2	0.0000	0.3197
c27353.graph_c1	(AT)9	229	3	0.0938	0.4582
c18775.graph_c0	(AT)9	273	3	0.0357	0.2457
c22422.graph_c0	(AT)9	254	3	0.0313	0.0854
c19149.graph_c0	(ATA)7	265	3	0.1250	0.3750
c16594.graph_c0	(CA)11	242	3	0.0667	0.2688
c28852.graph_c0	(CAA)7	170	3	0.1875	0.3047
c26585.graph_c1	(CT)10	113	3	0.0323	0.2765
c24756.graph_c0	(CT)9	167	4	0.1563	0.5718
c9302.graph_c0	(CT)9	252	3	0.0313	0.2713
c21449.graph_c0	(CT)9	152	3	0.1613	0.2174
c17362.graph_c0	(GA)9	248	2	0.0385	0.0733
c25883.graph_c0	(T)17	263	2	0.0625	0.0587
c29169.graph_c0	(TA)10	204	3	0.0938	0.3688
c9589.graph_c1	(TA)9	205	3	0.0323	0.2765
c28613.graph_c0	(TC)10	255	3	0.1290	0.2340
c20576.graph_c0	(TC)10	233	4	0.0400	0.2356
c19506.graph_c0	(TCA)7	238	3	0.0313	0.2125
c9818.graph_c0	(TCTA)5	108	4	0.1563	0.2557
c19643.graph_c0	(TTC)7	251	3	0.1250	0.3740

^a^Number of observed alleles.

^b^Number of Expected heterozygosity.

^c^Polymorphic information content.

**Fig 3:**
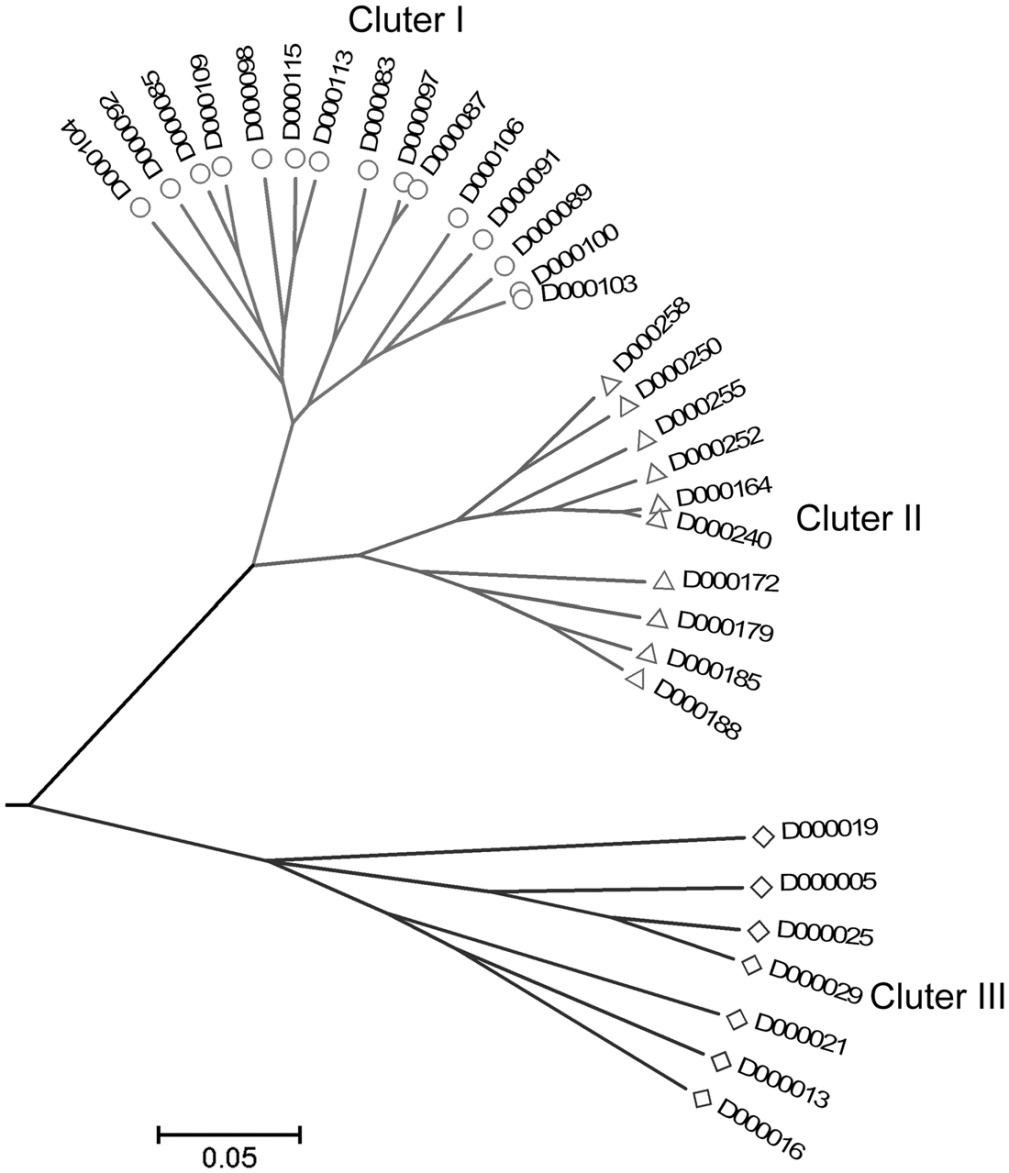
UPGMA dendrogram based on share allele distance showing genetic relationships among 32 rice bean accessions.

A UPGMA dendrogram based on shared allele distances clearly revealed three major clusters among 32 rice bean accessions (Fig 3). Cluster I consisted of accessions from Shandong province, in East China. Cluster II consisted of accessions from Hubei province, in Central China. Cluster III consisted of accessions from Shanxi province, in North China. These results showed an association between the genetic relationship of the rice bean and geographical origin.

## Discussion

In *Vigna* plants, whole genome and transcriptome sequence of mung bean [6, 22] and adzuki bean [23–25] has been reported, and the transcriptome sequence of cowpea [26] has also been reported. However, no genome or transcriptome sequences of rice bean have been developed until now and they have only been developed from mung bean [5] and adzuki bean [1]. In this study, we were obtained about 26 million clean reads from the rice bean transcriptome which assembled into 71,929 unigenes and 122,645 individual transcripts. The N50 length of the unigenes was 1,677 bp with an average length of 986 bp of rice bean. The length longer than those reported for the transcriptome of mung bean (874 bp and N50 = 1,563 bp) [6], common bean (813 bp and N50 = 1,449 bp) [27], and chickpea (1,065 bp and N50 = 1,653 bp) [28]. This result demonstrated the high quality of our rice bean transcriptome sequences.

*De novo* transcriptional sequencing is a rapid and cost-effective method for developing microsatellite markers [29, 30] and is a specifically a promising method for marker analysis in species with no reference genome [31–35]. Here, we showed that transcriptome sequencing is a very useful tool for unigene discovery and marker development in rice bean. To the best of our knowledge, this is the first large-scale development of SSR markers, and we have developed a total of 3,011 genic SSR markers from the 3,868 SSR-containing unigene sequences in this study.

In this study, the most abundant SSR motifs were the mono- (47.3%) and di-nucleotide (26.8%) repeats, followed by tri- (24.7%), tetra- (0.8%), penta- (0.2%) and hexa- (0.1%) in rice bean transcriptomes. Similar results have been reported in other related legume species, including mung bean [6], adzuki bean [23,24], cowpea [26] and chickpea [10]. The proportion of di- and tri-nucleotide repeats in rice bean transcript sequences was also very similar to those reported in mung bean, in which the proportion of di- and tri-nucleotide repeats was 26.8% and 24.7%, respectively [6].

Previous studies have estimated that, the number of AG motifs is 3,500 and 1,116, which account for 21.2% of the di-nucleotide motifs in adzuki bean [23] and 21.2% of the di-nucleotide motifs in mung bean [6], respectively. In this study, the number of AG motifs is 189 in rice bean transcript sequences, which accounts for 18.2% of di-nucleotide motifs. These data suggest that the AG motifs are abundant marker resources in the transcript sequences of *Vigna*. The most common di-nucleotide repeats were AG/CT (30.3%), followed by AT/TA (17.6%) and AC/GT (3.5%) in this study. The most general tri-nucleotide repeats were AAG/CTT (8.1%), followed by AAT/ATT (4.2%) and ACC/GGT (4.0%). Similar results have been were previously reported in other legumes such as adzuki bean [23, 24, 36] and mung bean [6], common bean [37], and faba bean [38].

Compared to the EST-SSRs developed for other *Vigna* crops including mung bean [6] and cowpea [26], the discriminating power as determined by the PIC value of the rice bean EST-SSRs with an average of 0.29 developed in this study, was less than that of mung bean EST-SSRs (average 0.34) [6] and cowpea (average 0.53) [26]. The low PIC values of our EST-SSRs suggested that the genetic sequences used for developing these markers were highly conserved in the rice bean germplasms used in this study. Nevertheless, polymorphic genic SSRs classified the rice bean germplasms of different geographical origins, and germplasms from the same province were clustered together by the genic SSRs (Fig 3). This finding suggested that rice bean cultivars in China were selected and had improved for a long time in specific environments, and thus those cultivars specifically adapted to such environments. However, due to the lack of pedigree information of the rice bean germplasms used in this study, we were not able to identify a genetic relationship among rice bean accessions in each sub-cluster. Previous studies have indicated that the distribution of rice bean accessions that originate from China within each cluster correlates with the geographical regions of their sampling origin [5], which is closely in agreement with our study.

In summary, a large number of high-quality unigene sequences have been developed from rice bean via next-generation sequencing and a large number of genic SSRs have identified in our study. We designed a total of 3,011 SSR primer pairs from the unigene sequences which need to be further validated for amplification. Indeed, random marker validation showed that only 43 (14.3%) of the genic SSR markers were amplifiable with clear and expected product sizes, and 23 (53.5%) showed polymorphisms among 32 rice been accessions. The low amplifiable rate and low polymorphism rate in our study may stem from (i) a large intron between primers, (ii) unrecognized intron splice sites that can disrupt priming sites, and (iii) sequencing error.

Although the amplifiable markers showed low polymorphism in the rice bean germplasm, they were useful in revealing the genetic relationships of the germplasm. Our study is the first study to develop the genic SSRs for rice bean using the next-generation sequencing technology. Our results demonstrate that *in silico* SSR marker development by transcriptome sequencing using NGS can be used to detect (or allow detecting) genic SSRs for crops with low genomic resources.

## Supporting Information

S1 Table Germplasm accessions used in this diversity study of rice bean. (DOC)

S2 Table List of the primer pairs designed for the 3,868 SSR loci. (XLS)

S3 Table Primer sequences of 300 genic SSR markers used for marker validation. (XLS)

S4 Table The putative proteins identified by BLASTX of 23 unigene sequences containing polymorphic genic SSRs. (DOC)
