# Supplementary material for: Development of Gene-Based SSR Markers in Rice Bean (Vigna umbellata L.) Based on Transcriptome Data
Source: PLoS One. 2016 Mar 7;11(3):e0151040. doi: 10.1371/journal.pone.0151040 (PMC4780709; doi:10.1371/journal.pone.0151040)
Supplement: S1 Table — (DOC) [file pone.0151040.s001.doc]

**S1 Table. Germplasm accessions used in this diversity study of rice bean.**

| **Serial No.** | **Accession number** | **Province of origin** | **Habit of growth** | **Seed coat color** |
| --- | --- | --- | --- | --- |
| 1 | D0000005 | Shanxi | Sprawl | Red |
| 2 | D0000013 | Shanxi | Sprawl | Red |
| 3 | D0000016 | Shanxi | Sprawl | Yellow |
| 4 | D0000019 | Shanxi | Sprawl | Yellow |
| 5 | D0000021 | Shanxi | Sprawl | Yellow |
| 6 | D0000025 | Shanxi | Sprawl | Yellow |
| 7 | D0000029 | Shanxi | Sprawl | Yellow |
| 8 | D0000083 | Shandong | Sprawl | Red |
| 9 | D0000085 | Shandong | Sprawl | Yellow |
| 10 | D0000087 | Shandong | Sprawl | Yellow |
| 11 | D0000089 | Shandong | Sprawl | Yellow |
| 12 | D0000091 | Shandong | Sprawl | Yellow |
| 13 | D0000092 | Shandong | Sprawl | Yellow |
| 14 | D0000097 | Shandong | Sprawl | Yellow |
| 15 | D0000098 | Shandong | Sprawl | Yellow |
| 16 | D0000100 | Shandong | Sprawl | Yellow |
| 17 | D0000103 | Shandong | Sprawl | Yellow |
| 18 | D0000104 | Shandong | Sprawl | Yellow |
| 19 | D0000106 | Shandong | Sprawl | Yellow |
| 20 | D0000109 | Shandong | Sprawl | Yellow |
| 21 | D0000113 | Shandong | Sprawl | Yellow |
| 22 | D0000115 | Shandong | Sprawl | Yellow |
| 23 | D0000164 | Hubei | Sprawl | Red |
| 24 | D0000172 | Hubei | Sprawl | Red |
| 25 | D0000179 | Hubei | Sprawl | Red |
| 26 | D0000185 | Hubei | Sprawl | Red |
| 27 | D0000188 | Hubei | Sprawl | Red |
| 28 | D0000240 | Hubei | Sprawl | Yellow |
| 29 | D0000250 | Hubei | Sprawl | Yellow |
| 30 | D0000252 | Hubei | Sprawl | Yellow |
| 31 | D0000255 | Hubei | Sprawl | Yellow |
| 32 | D0000258 | Hubei | Sprawl | Pinto |

Note: these accessions were from National Center for Crop Germplasm Resources Preservation of China.
