# Supplementary material for: Development of Gene-Based SSR Markers in Rice Bean (Vigna umbellata L.) Based on Transcriptome Data
Source: PLoS One. 2016 Mar 7;11(3):e0151040. doi: 10.1371/journal.pone.0151040 (PMC4780709; doi:10.1371/journal.pone.0151040)
Supplement: S4 Table — (DOC) [file pone.0151040.s004.doc]

**S4 Table. The putative proteins identified by BLASTX of 23 unigene sequences containing polymorphic genic SSRs.**

| **SSR locus** | **Organism**  **(****Glycine max)** | **GenBank (Accession No.)** | **Organism**  **([Vigna angularis var. angularis](http://blast.ncbi.nlm.nih.gov/Blast.cgi#alnHdr_965656162))** | **GenBank (Accession No.)** |
| --- | --- | --- | --- | --- |
| c9711.graph_c0 | [T](http://blast.ncbi.nlm.nih.gov/Blast.cgi#alnHdr_955354281)ranscription factor TCP4-like (LOC100811837) | [XM_006592389.2](http://www.ncbi.nlm.nih.gov/nucleotide/955354281?report=genbank&log$=nucltop&blast_rank=2&RID=96S5135B01R) | [C](http://blast.ncbi.nlm.nih.gov/Blast.cgi#alnHdr_965601945)hromosome 6, almost complete sequence | [AP015039.1](http://www.ncbi.nlm.nih.gov/nucleotide/965601945?report=genbank&log$=nucltop&blast_rank=1&RID=98MEWCZ4013) |
| c19803.graph_c1 | No hit | – | No hit | – |
| c21640.graph_c0 | No hit | – | [C](http://blast.ncbi.nlm.nih.gov/Blast.cgi#alnHdr_965615722)hromosome 11, almost complete sequence | [AP015044.1](http://www.ncbi.nlm.nih.gov/nucleotide/965615722?report=genbank&log$=nucltop&blast_rank=1&RID=98MTE737013) |
| c19719.graph_c0 | [C](http://blast.ncbi.nlm.nih.gov/Blast.cgi#alnHdr_955379595)haperone protein ClpB-like (LOC100809965) | [XM_003549457.3](http://www.ncbi.nlm.nih.gov/nucleotide/955379595?report=genbank&log$=nucltop&blast_rank=2&RID=96U8MCDU01R) | [C](http://blast.ncbi.nlm.nih.gov/Blast.cgi#alnHdr_965656162)hromosome 1, almost complete sequence | [AP015034.1](http://www.ncbi.nlm.nih.gov/nucleotide/965656162?report=genbank&log$=nucltop&blast_rank=1&RID=96U8MCDU01R) |
| c27353.graph_c1 | No hit | – | [C](http://blast.ncbi.nlm.nih.gov/Blast.cgi#alnHdr_965610582)hromosome 9, almost complete sequence | [AP015042.1](http://www.ncbi.nlm.nih.gov/nucleotide/965610582?report=genbank&log$=nucltop&blast_rank=1&RID=96U9TJZC01R) |
| c18775.graph_c0 | No hit | – | [C](http://blast.ncbi.nlm.nih.gov/Blast.cgi#alnHdr_965668308)hromosome 4, almost complete sequence | [AP015037.1](http://www.ncbi.nlm.nih.gov/nucleotide/965668308?report=genbank&log$=nucltop&blast_rank=1&RID=96UJUSA501R) |
| c22422.graph_c0 | No hit | – | [C](http://blast.ncbi.nlm.nih.gov/Blast.cgi#alnHdr_965604228)hromosome 7, almost complete sequence | [AP015040.1](http://www.ncbi.nlm.nih.gov/nucleotide/965604228?report=genbank&log$=nucltop&blast_rank=1&RID=96UKME1Y01R) |
| c19149.graph_c0 | [H](http://blast.ncbi.nlm.nih.gov/Blast.cgi#alnHdr_955395344)omeotic protein knotted-1-like (LOC100800822), mRNA | [XM_003556915.3](http://www.ncbi.nlm.nih.gov/nucleotide/955395344?report=genbank&log$=nucltop&blast_rank=2&RID=96UPZDR501R) | [C](http://blast.ncbi.nlm.nih.gov/Blast.cgi#alnHdr_965664935)hromosome 3, almost complete sequence | [AP015036.1](http://www.ncbi.nlm.nih.gov/nucleotide/965664935?report=genbank&log$=nucltop&blast_rank=1&RID=96UPZDR501R) |
| c16594.graph_c0 | No hit | – | [C](http://blast.ncbi.nlm.nih.gov/Blast.cgi#alnHdr_965601945)hromosome 6, almost complete sequence | [AP015039.1](http://www.ncbi.nlm.nih.gov/nucleotide/965601945?report=genbank&log$=nucltop&blast_rank=1&RID=96UVBMNE01R) |
| c28852.graph_c0 | Hypothetical protein (PHAVU_006G200900g) mRNA, | XM_007148289.1 | [C](http://blast.ncbi.nlm.nih.gov/Blast.cgi#alnHdr_965601945)hromosome 6, almost complete sequence | [AP015039.1](http://www.ncbi.nlm.nih.gov/nucleotide/965601945?report=genbank&log$=nucltop&blast_rank=1&RID=96UYY2GT01R) |
| c26585.graph_c1 | [C](http://blast.ncbi.nlm.nih.gov/Blast.cgi#alnHdr_144925085)lone gmp1-120k16, complete sequence | [AC188370.9](http://www.ncbi.nlm.nih.gov/nucleotide/144925085?report=genbank&log$=nucltop&blast_rank=38&RID=96UZR7UZ01R) | [C](http://blast.ncbi.nlm.nih.gov/Blast.cgi#alnHdr_965668308)hromosome 4, almost complete sequence | [AP015037.1](http://www.ncbi.nlm.nih.gov/nucleotide/965668308?report=genbank&log$=nucltop&blast_rank=40&RID=96UZR7UZ01R) |
| c24756.graph_c0 | No hit | – | [C](http://blast.ncbi.nlm.nih.gov/Blast.cgi#alnHdr_965607001)hromosome 8, almost complete sequence | [AP015041.1](http://www.ncbi.nlm.nih.gov/nucleotide/965607001?report=genbank&log$=nucltop&blast_rank=1&RID=96VF180M01R) |
| c9302.graph_c0 | No hit | – | [C](http://blast.ncbi.nlm.nih.gov/Blast.cgi#alnHdr_965656162)hromosome 1, almost complete sequence | [AP015034.1](http://www.ncbi.nlm.nih.gov/nucleotide/965656162?report=genbank&log$=nucltop&blast_rank=1&RID=96VN4XES01R) |
| c21449.graph_c0 | No hit | – | [C](http://blast.ncbi.nlm.nih.gov/Blast.cgi#alnHdr_965613050)hromosome 10, almost complete sequence | [AP015043.1](http://www.ncbi.nlm.nih.gov/nucleotide/965613050?report=genbank&log$=nucltop&blast_rank=1&RID=96VYK29G01R) |
| c17362.graph_c0 | No hit | – | [C](http://blast.ncbi.nlm.nih.gov/Blast.cgi#alnHdr_965601945)hromosome 6, almost complete sequence | [AP015039.1](http://www.ncbi.nlm.nih.gov/nucleotide/965601945?report=genbank&log$=nucltop&blast_rank=1&RID=96W2YM9S01R) |
| c25883.graph_c0 | No hit | – | [C](http://blast.ncbi.nlm.nih.gov/Blast.cgi#alnHdr_965604228)hromosome 7, almost complete sequence | [AP015040.1](http://www.ncbi.nlm.nih.gov/nucleotide/965604228?report=genbank&log$=nucltop&blast_rank=1&RID=96W7ANW401R) |
| c29169.graph_c0 | No hit | – | [C](http://blast.ncbi.nlm.nih.gov/Blast.cgi#alnHdr_965607001)hromosome 8, almost complete sequence | |  | [AP015041.1](http://www.ncbi.nlm.nih.gov/nucleotide/965607001?report=genbank&log$=nucltop&blast_rank=1&RID=96W86Z8H01R) | | --- | --- | |
| c9589.graph_c1 | No hit | – | [C](http://blast.ncbi.nlm.nih.gov/Blast.cgi#alnHdr_965607001)hromosome 8, almost complete sequence | [AP015041.1](http://www.ncbi.nlm.nih.gov/nucleotide/965607001?report=genbank&log$=nucltop&blast_rank=1&RID=96WAJTK501R) |
| c28613.graph_c0 | Putative uncharacterized protein DDB_G0288537(LOC100783978) | [XM_014761850.1](http://www.ncbi.nlm.nih.gov/nucleotide/955339794?report=genbank&log$=nucltop&blast_rank=4&RID=98M7K0P7016) | [C](http://blast.ncbi.nlm.nih.gov/Blast.cgi#alnHdr_965656162)hromosome 1, almost complete sequence | |  | [AP015034.1](http://www.ncbi.nlm.nih.gov/nucleotide/965656162?report=genbank&log$=nucltop&blast_rank=1&RID=98M7K0P7016) | | --- | --- | |
| c20576.graph_c0 | No hit | – | [C](http://blast.ncbi.nlm.nih.gov/Blast.cgi#alnHdr_965656162)hromosome 1, almost complete sequence | |  | [AP015034.1](http://www.ncbi.nlm.nih.gov/nucleotide/965656162?report=genbank&log$=nucltop&blast_rank=1&RID=98M46K6C016) | | --- | --- | |
| c19506.graph_c0 | No hit | – | [C](http://blast.ncbi.nlm.nih.gov/Blast.cgi#alnHdr_965615722)hromosome 11, almost complete sequence | [AP015044.1](http://www.ncbi.nlm.nih.gov/nucleotide/965615722?report=genbank&log$=nucltop&blast_rank=1&RID=98M2397X016) |
| c9818.graph_c0 | No hit | – | [C](http://blast.ncbi.nlm.nih.gov/Blast.cgi#alnHdr_965668308)hromosome 4, almost complete sequence | |  | [AP015037.1](http://www.ncbi.nlm.nih.gov/nucleotide/965668308?report=genbank&log$=nucltop&blast_rank=82&RID=98KYDCA2016) | | --- | --- | |
| c19643.graph_c0 | No hit | – | [C](http://blast.ncbi.nlm.nih.gov/Blast.cgi#alnHdr_965599522)hromosome 5, almost complete sequence | [AP015038.1](http://www.ncbi.nlm.nih.gov/nucleotide/965599522?report=genbank&log$=nucltop&blast_rank=1&RID=98KTNC75016) |
